# Niacin Supplementation Alleviates TCIPP-Induced Lung Injury via Inhibition of the NF-κB Signaling Pathway

**DOI:** 10.3390/antiox15010085

**Published:** 2026-01-08

**Authors:** Meiyu Zhou, Xiaoyu Gao, Ruiyang Tian, Taiyu Gu, Ziwei Dong, Wenjun Shi, Tianyao Mao, Zhengdong Zhang, Haiyan Chu

**Affiliations:** Department of Environmental Genomics, School of Public Health, Nanjing Medical University, 101 Longmian Avenue, Jiangning District, Nanjing 211166, China; zhoumeiyu707@163.com (M.Z.); 18943530693@163.com (X.G.); tianruiyangll@163.com (R.T.); gutaiyu10@gmail.com (T.G.); ziwei2025@163.com (Z.D.); wenjunshi2000@163.com (W.S.); 18912528509@163.com (T.M.)

**Keywords:** Tris(1-chloro-2-propyl) phosphate (TCIPP), niacin, oxidative stress, lung injury, NF-κB signaling pathway

## Abstract

Tris(1-chloro-2-propyl) phosphate (TCIPP) is an emerging environmental pollutant associated with adverse respiratory effects, yet whether niacin has a protective effect on lung function remains unclear. Data from 1031 participants in the 2011–2012 National Health and Nutrition Examination Survey (NHANES) were analyzed using multiple linear regression to assess associations between urinary bis(1,3-dichloro-2-propyl) phosphate (BCIPP), dietary niacin intake, and pulmonary function. Animal models were established to investigate TCIPP-induced lung injury and the protective effects of niacin. Lung injury was assessed by histopathology, lung function, inflammation, and oxidative stress-related indicators. Comparative Toxicogenomics Database (CTD), molecular docking, and Western blot were performed to explore underlying mechanisms. Higher urinary BCIPP concentration was associated with reduced lung function, whereas higher dietary niacin intake was associated with improved lung function. Notably, BCIPP levels showed positive associations between dietary niacin intake and FEV_1_ [β (95% CI) = 0.11 (0.06, 0.16), *p_adj_* < 0.001] and FVC [β (95% CI) = 0.09 (0.05, 0.13), *p_adj_* < 0.001] in males with lower BCIPP exposure. In male mice, TCIPP exposure caused dose-dependent lung injury, inflammation, and oxidative stress, while niacin supplementation alleviated lung damage, improved lung function, and restored antioxidant defenses by inhibiting NF-κB phosphorylation. Niacin supplementation alleviated TCIPP-induced lung injury in males by inhibiting oxidative stress and NF-κB activation, suggesting niacin as a potential nutritional strategy to improve lung function.

## 1. Introduction

Respiratory diseases, such as chronic obstructive pulmonary disease (COPD), asthma, and interstitial lung disease, remain a major global health burden with high morbidity and mortality [1]. Lung function decline is an early and important manifestation of respiratory diseases [2]. Impaired lung function accelerates disease progression, aggravates symptoms, and increases the risk of systemic complications, including cardiovascular and metabolic disorders [3]. Lung function testing is one of the most sensitive and valuable tools for detecting early, asymptomatic abnormalities, allowing prevention and intervention before clinical symptoms appear [4].

The etiology of lung function impairment is complex and remains unclear. Environmental pollutants are important contributing factors [5]. Tris(1-chloro-2-propyl) phosphate (TCIPP) has emerged as a pervasive environmental pollutant due to its extensive use in household and industrial products [6]. Because TCIPP is physically incorporated into polymers, it is prone to release into the environment through volatilization, leaching, and abrasion [7], particularly in indoor air and dust [6,8,9,10,11]. Therefore, inhalation exposure is one of the most significant pathways for human exposure to TCIPP [12]. Accumulating evidence indicates that TCIPP possesses various toxic effects, including neurotoxicity [13], endocrine disruption [14,15], and immunotoxicity [16]. Notably, bis(1,3-dichloro-2-propyl) phosphate (BCIPP), the primary metabolite of TCIPP, has been associated with reduced lung function [17], and BCIPP levels vary across specific population subgroups [18]. Studies have shown that internal exposure to environmental pollutants may differentially affect pulmonary outcomes among individuals and subgroups [19]. These findings suggest that both internal exposure levels and susceptibility to TCIPP may differ according to sex and other individual characteristics.

While a comprehensive understanding of the effect mechanisms associated with TCIPP remains elusive, oxidative stress is widely considered a central mechanism. Meng et al. revealed that TCIPP induces oxidative stress in non-small cell lung cancer cell lines by inducing excessive production of reactive oxygen species (ROS), thereby participating in lung injury [20]. Additionally, TCIPP can affect the viability of human lung cancer A549 cells and the mitochondrial membrane potential to increase lung damage [21]. In addition, long-term exposure to tobacco smoke and environmental pollutants enhances inflammation and oxidative stress, ultimately causing irreversible damage to the lungs [22,23]. Oxidative damage leads to lipid peroxidation, protein modification, and DNA lesions, which promote tissue injury, airway remodeling, and impaired lung function [24]. Importantly, early oxidative stress and inflammation are recognized as initial events preceding chronic respiratory diseases [25]. These studies highlight the significance of oxidative stress in the development of pollutant-induced respiratory diseases, with activation of the nuclear factor kappa B (NF-κB) signaling pathway acting as a critical molecular link between oxidative stress and pollutant-induced inflammatory lung injury [26].

It is noteworthy that respiratory diseases exhibit robust correlations with various oxidative and antioxidant dietary factors. The polyphenols, antioxidants, and dietary fibers in fruits and vegetables can counteract the inflammation and oxidative stress caused by PM_2.5_ and NOx exposure, thereby alleviating the progression of chronic obstructive pulmonary disease (COPD) [27]. Given the ubiquitous presence of TCIPP in the environment and its central role in inducing oxidative stress-related lung damage, dietary interventions targeting oxidative pathways offer a promising strategy to mitigate its adverse effects. Niacin (vitamin B3), a vital nutrient, has garnered interest due to its potent antioxidant and anti-inflammatory properties [28]. Niacin is known to enhance cellular redox balance, modulate inflammatory signaling pathways, and protect lung tissue from oxidative insults [29]. Mechanistically, niacin has been reported to influence oxidative stress-related pathways, including the NF-κB signaling cascade, thereby attenuating inflammatory amplification [30]. Epidemiological studies have demonstrated that niacin intake is linked to improved lung function, with higher levels of niacin intake associated with increased forced expiratory volume (FEV_1_) and reduced airway impairment [31,32]. A large-scale study in Chinese populations found the protective effect of fish consumption on COPD, which is rich in niacin [33]. Niacin improves redox homeostasis, modulates inflammatory signaling, and has been associated with better lung function in epidemiological studies [34]. However, evidence on whether niacin can counteract lung damage induced by organophosphate flame retardants such as TCIPP remains limited.

Therefore, in this study, we integrated epidemiological data analysis and animal models to explore the relationship between urinary BCIPP levels, dietary niacin intake, and lung injury. We used data from the 2011–2012 National Health and Nutrition Examination Survey (NHANES) cycle to evaluate the association of BCIPP with lung function, investigate its potential interaction with dietary niacin intake, and explore sex-specific susceptibility via stratified analyses. Furthermore, a mouse model of TCIPP-induced lung injury was developed in C57BL/6 mice to investigate the effects of niacin supplementation and the associated molecular mechanisms, particularly those involving oxidative stress. Therefore, we performed this study to systematically elucidate the interplay between individual susceptibility, dietary factors, and mechanistic pathways underlying TCIPP-induced lung injury.

## 2. Materials and Methods

### 2.1. Study Population

We obtained the data from the 2011–2012 cycle of the NHANES, a publicly available, nationally representative cross-sectional survey conducted by the National Center for Health Statistics (NCHS) to assess the health and nutritional status of the non-institutionalized U.S. population. This cycle was selected as it represents the sole survey cycle that simultaneously provides data on urinary OPFRs metabolites and standardized spirometry-based lung function measurements. All NHANES data are publicly accessible through the NHANES database (https://wwwn.cdc.gov/nchs/nhanes/Default.aspx, accessed on 30 August 2024). The NHANES study protocol was approved by the NCHS Ethics Review Board, and all participants provided written informed consent prior to participation. Because the present study exclusively involved secondary analysis of de-identified, publicly available NHANES data, no additional ethical approval was required. All procedures were conducted in accordance with the Declaration of Helsinki. Among the 9756 individuals initially enrolled, 57 pregnant participants and 7677 individuals without urinary BCIPP data were excluded, leaving 2022 participants with valid BCIPP measurements. After applying the inclusion criteria, 1173 participants were retained. Further exclusions included 269 individuals with missing or invalid lung function measurements, 580 individuals under 20 years of age, 64 participants with missing dietary niacin intake data, and 78 with implausible energy intakes (<800 or >4200 kcal/day for males; <500 or >3500 kcal/day for females). Finally, a total of 1031 participants were included in the study (Figure S1).

### 2.2. Determination of Urinary BCIPP Concentration

Urine samples were collected when participants entered the examination center, following the analytic protocols of NHANES. The concentration of BCIPP was quantified using solid-phase extraction coupled with high-performance liquid chromatography-tandem mass spectrometry (SPE-HPLC-MS/MS). Detailed detection methods for m-OPFRs in human and mouse samples are provided in the Supporting Information. According to NHANES laboratory analytic guidelines, BCIPP concentrations below the limit of detection (LOD, 0.10 μg/L) were imputed as the LOD divided by the square root of two (LOD/√2). In the present study, the detection rate of urinary BCIPP was 54.70% (Table S1).

### 2.3. Dietary Niacin Intake Data

Dietary niacin intake data estimated using the NHANES Computer-Assisted Dietary Interview System were collected and recorded based on the 24 h prior to the interview. Dietary niacin intake was calculated using the Food and Nutrient Database for Dietary Studies (FNDDS). Given its ability to capture detailed food consumption data, the 24 h dietary recall method was employed over the food frequency questionnaire to ensure a more precise assessment of nutrient intake. The dietary niacin intake data from NHANES 2011–2012 were extracted (Table 1). The methodologies applied in dietary data collection are detailed in the NHANES Dietary Interviewers Procedure Manuals.

### 2.4. Lung Function Index Assessment

Pulmonary function was assessed in human participants and mice. In humans, spirometry was performed in the standing position, unless physically limited, with measurements of forced expiratory volume in one second (FEV_1_), forced vital capacity (FVC), peak expiratory flow (PEF), forced expiratory flow at 25–75% of FVC (FEF_25–75%_), and the FEV_1_/FVC ratio, with a maximum of eight tests conducted to ensure acceptable results. Only participants with FEV_1_ and FVC rated A or B were included. As described in previous studies [35], following anesthesia and tracheal intubation, mouse pulmonary function was assessed using the Forced Maneuvers System (EMMS, Hants, UK). The system was calibrated daily before testing. Standardized spirometric maneuvers were conducted to measure lung function through indices reflecting airflow dynamics and lung volumes. Parameters indicative of airway patency and airflow limitation included peak expiratory flow (PEF) and maximal mid-expiratory flow (MMEF), whereas those related to lung capacity, compliance, and structural characteristics comprised inspiratory capacity (IC), forced vital capacity (FVC), residual volume (REV), functional residual capacity (FRC), and total lung capacity (TLC). For each mouse, three consecutive, technically acceptable maneuvers were recorded for each parameter. The mean value of the three replicates was calculated and used for subsequent statistical analyses. Mice were euthanized immediately after pulmonary function testing for tissue collection.

### 2.5. The Association Between BCIPP/Niacin and Lung Function

Weighted linear models were employed to explore the interactions of BCIPP and niacin on lung function. To illustrate the complex sampling procedure, weights were taken into account in the analysis (BCIPP weight WTSSBG2Y), using three progressively adjusted models. Detailed information for covariates is provided in the Supporting Information.

### 2.6. Animal Models

A total of 120 SPF-grade C57BL/6 mice, aged 6 weeks, were purchased from the Animal Core Facility of Nanjing Medical University (Nanjing, China). All animal procedures were carried out in accordance with the regulations of the Laboratory Animal Center of Nanjing Medical University and conformed to accepted animal ethics guidelines (Ethics number: IACUC-2411024; Approval Date: 8 November 2024). We firstly constructed a high-exposure group (100 × RfD) TCIPP mice exposure model with significant lung injury, which was based on the United States Environmental Protection Agency reference dose (RfD) for TCIPP (0.01 mg/kg/day) [36]. According to these findings, we further established the research design of animal models. Study 1: eighty mice were randomly assigned to eight groups (*N* = 10 per group, balanced by sex). As follows: control group, RfD group (0.01 mg/kg/day), 10 × RfD group (0.1 mg/kg/day), and 100 × RfD group (1 mg/kg/day). A 4-week exposure model to TCIPP (Macklin, Shanghai, China) was established via intratracheal instillation, a well-established and reliable approach in inhalation toxicology for delivering inhaled contaminants to the respiratory tract and evaluating pollutant-induced pulmonary toxicity [37]. Study 2: Forty male mice were randomly divided into four groups (*N* = 10 per group): control group, TCIPP exposure group (0.1 mg/kg/day), niacin treatment group (300 mg/kg/day, Macklin, Shanghai, China), and TCIPP exposure followed by niacin intervention group (co-treatment with 0.1 mg/kg/day TCIPP and 300 mg/kg/day niacin). TCIPP was delivered by intratracheal instillation for 4 weeks, while niacin (Macklin, China) was administered via oral gavage 2 h after TCIPP exposure. The niacin dose ranged from 200 to 400 mg/kg/day according to the previous study [38]. This study included 300 mg/kg/day in the animal model. All mice were euthanized within 24 h after the final treatment, and lung tissues were collected for subsequent analyses. Histological analyses were conducted to evaluate lung morphology and injury, including semi-quantitative pathological scoring of lung tissues, quantification of inflammatory cell infiltration, mean linear intercept (MLI), destruction index (DI), airway wall thickness, and airway wall area. All histological and quantitative assessments were performed by investigators blinded to experimental group allocation. Enzyme-linked immunosorbent assay (ELISA) was used to quantify inflammatory cytokines interleukin-6 (IL-6) and transforming growth factor-β1 (TGF-β1) in lung tissues. Oxidative stress status was assessed by measuring superoxide dismutase (SOD) activity, glutathione peroxidase (GSH-Px) activity, and malondialdehyde (MDA) content. Western blot analysis was performed to evaluate modulation of the NF-κB signaling pathway in lung tissues. Detailed experimental procedures are provided in the Supporting Information.

### 2.7. Niacin-Related Gene and Phenotypic Data Collection

Candidate genes and Pathways related to Cl-OPFRs were collected by querying the CTD (http://ctdbase.org, accessed on 10 June 2025) using the keywords “niacin”under the “Genes” and “Pathways” data-tabs, respectively. The target phenotypes were based on the intersection of the phenotypes obtained by the Kyoto Encyclopedia of Genes and Genomes (KEGG) pathway enrichment analysis of the candidate genes and relevant phenotypes in the CTD.

### 2.8. Molecular Docking Analysis of Niacin with Oxidative Stress-Associated Proteins

To investigate the interaction between niacin and oxidative stress-associated proteins, five target proteins were selected based on the top-ranking genes identified in the KEGG pathway enrichment analysis of oxidative stress-related pathways. The 3D structure of niacin was obtained from the PubChem database (https://pubchem.ncbi.nlm.nih.gov, accessed on 20 April 2025), and the corresponding protein structures were downloaded from the Protein Data Bank (PDB, https://www.rcsb.org, accessed on 20 April 2025). Protein structures were preprocessed using PyMOL (version 3.1.0) by removing water molecules and any co-crystallized ligands. AutoDockTools (version 1.5.7) was used to prepare the proteins by adding polar hydrogens, calculating Gasteiger charges, and identifying the active binding pockets. The ligand (niacin) was also converted into the appropriate docking format using the same software. Molecular docking was performed between niacin and five target proteins. Binding affinities (binding energies) were calculated for each docking pair, and the protein with the strongest binding affinity to niacin was identified. The docking conformations of niacin with each protein were visualized using PyMOL (version 3.1.0).

### 2.9. Statistical Analyses

Continuous variables were summarized as mean ± standard deviation (SD), and categorical variables were expressed as numbers and percentages. Urinary BCIPP concentrations exhibited a right-skewed distribution and were therefore natural log-transformed prior to analysis; geometric means and geometric standard deviations were reported for descriptive purposes. For descriptive comparisons across groups, continuous variables were compared using one-way analysis of variance (ANOVA), followed by Tukey’s post hoc multiple comparisons test when appropriate, while categorical variables were compared using χ^2^ tests. Associations between urinary BCIPP concentrations, dietary niacin intake, and lung function parameters were examined using survey-weighted linear regression models to account for the complex sampling design of NHANES. For analyses involving multiple comparisons, false discovery rate (FDR) correction was applied as a sensitivity analysis. All statistical analyses were performed using R software (version 4.2.1). A two-sided *p* value < 0.05 was considered statistically significant.

## 3. Results

### 3.1. Basic Characteristics of Study Participants

As shown in Table S2, a total of 1031 participants (49.85% male, 50.15% female) with a mean age of 46.99 ± 16.12 years were included. No significant differences were observed between males and females in age, race distribution, or family poverty-income ratio. However, significant sex differences were observed in education level, body mass index, physical activity, serum cotinine concentrations, and urinary creatinine levels.

The geometric mean urinary BCIPP concentration was 0.15 ± 2.55 µg/L and 1.50 ± 2.65 ng/g creatinine, with no significant difference between sexes. Dietary niacin intake was significantly higher in males (28.75 ± 14.35 mg/d) compared to females (20.51 ± 9.59 mg/d, *p* < 0.001). Lung function measures indicated an average FEV_1_ of 7.96 ± 0.31 mL, FVC of 8.21 ± 0.29 mL, PEF of 8.94 ± 0.31 mL/s, FEF_25–75%_ of 7.85 ± 0.54 mL/s, and the FEV_1_/FVC ratio was 0.58 ± 0.05. There are significant differences in lung function indicators between males and females (all *p* < 0.001, Table 1). These findings emphasize the potential sex differences in individuals in pulmonary health and dietary intake.

### 3.2. Association of BCIPP and Niacin with Lung Function

#### 3.2.1. Independent Association Between BCIPP/Niacin and Lung Function

Table S3 presents the associations between urinary BCIPP concentrations, dietary niacin intake, and lung function parameters across three adjusted models. BCIPP was associated with decreased levels of FEV_1_ [β (95% CI) = −0.05 (−0.10, 0.00), *p* = 0.041] and PEF [β (95% CI) = −0.06 (−0.11, −0.01), *p* = 0.042] in Model 3. These associations did not remain statistically significant after FDR correction. In contrast, higher dietary niacin intake was positively associated with lung function, especially for FEV_1_ [β (95% CI) = 0.03 (0.00, 0.06), *p* = 0.009] and FVC (β (95% CI) = 0.04 (0.02, 0.06), *p* < 0.001), the association remained statistically significant after adjustment for multiple comparisons (*p_adj_* < 0.05).

#### 3.2.2. The Interaction Effects of BCIPP and Niacin on Lung Function

**Figure 1:**
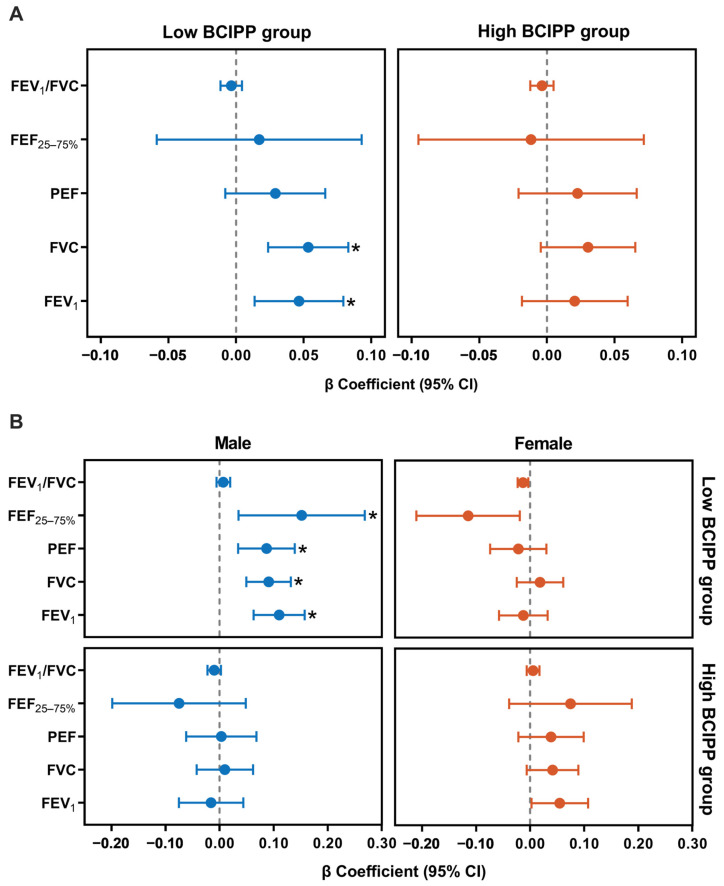
The interaction effects of BCIPP and dietary niacin intake on lung function. (**A**) Forest plot showed the association between niacin exposure and lung function indicators stratified by the concentration of urinary BCIPP, categorized into low and high groups based on the median BCIPP concentration. (**B**) Forest plot showed the association between niacin exposure and lung function indicators stratified by the median concentration of urinary BCIPP and gender. The results were adjusted for age, sex, race, family poverty-income ratio, educational level, BMI, physical activity, serum cotinine, and urinary creatinine. BCIPP, bis(1-chloro-2-propyl) phosphate; FEV_1_, forced expiratory volume first second; FVC, forced vital capacity; PEF, peak expiratory flow; FEF_25–75%_, forced expiratory flow at 25–75% of FVC. * indicates statistically significant associations after FDR correction (*p_adj_* < 0.05).

To assess the interaction, we stratified the analysis by BCIPP exposure, using the median urinary concentration as the cutoff to define low and high exposure groups. The associations between urinary BCIPP concentrations and lung function after stratification by BCIPP exposure levels (high vs. low). In the low-BCIPP exposure group, higher dietary niacin intake was significantly associated with improved lung function of individuals with FEV_1_ [β (95% CI) = 0.05 (0.01, 0.08), *p_adj_* = 0.029] and FVC [β (95% CI) = 0.05 (0.02, 0.08), *p_adj_* = 0.005, Figure 1A]. Stratification by sex showed the significant positive associations between niacin intake and FEV_1_ [β (95% CI) = 0.11 (0.06, 0.16), *p_adj_* < 0.001], FVC [β (95% CI) = 0.09 (0.05, 0.13), *p_adj_* < 0.001], PEF [β (95% CI) = 0.09 (0.04, 0.14), *p_adj_* = 0.009], and FEF_25–75%_ [β (95% CI) = 0.15 (0.04, 0.27), *p_adj_* = 0.046] in the low-BCIPP exposed males group. For females, we did not observe a similar association (Figure 1B).

### 3.3. TCIPP Exposure-Induced Lung Injury and Oxidative Stress

**Figure 2:**
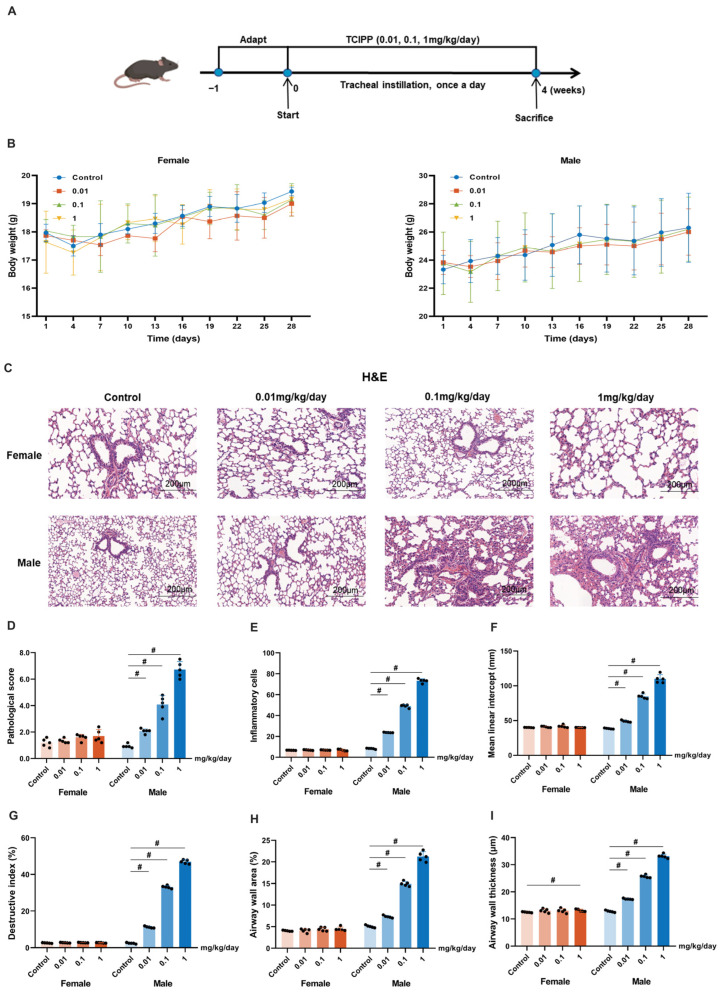
TCIPP exposure induces lung injury in Mice (*N* = 5 per group). (**A**) Experimental timeline showing the administration of TCIPP (0.01, 0.1, and 1 mg/kg/day) via tracheal instillation for 4 weeks. (**B**) Body weight changes in female and male mice over the treatment period. (**C**) Representative H&E-stained lung sections for female and male mice, illustrating the histopathological changes at multiple TCIPP doses, scale bars represent 200 µm. (**D**) Pathological scores of lung injuries. (**E**) Inflammatory cell counts. (**F**) Mean linear intercept. (**G**) Destructive index. (**H**) Airway wall area. (**I**) Airway wall thickness. TCIPP, Tris(2-chloroisopropyl) phosphate. Data are expressed as mean ± SEM; # *p* < 0.05, determined by one-way ANOVA followed by Tukey’s multiple comparisons test.

To further investigate the lung toxicity of TCIPP, the mouse models were established with intratracheal instillation of TCIPP at doses of 0.01, 0.1, and 1 mg/kg/day for 4 weeks (Figure 2A). No significant differences in body weight were observed among treated and control groups (Figure 2B). Urinary concentrations of BCIPP, the primary metabolite of TCIPP, increased dose-dependently in male mice. However, female mice showed the stable BCIPP concentration levels (0.07–0.18 µg/L or 303–650 ng/g creatinine), which were significantly lower than those in males (0.11–4.41 µg/L or 472–19,867 ng/g creatinine, Figure S2). In male mice, TCIPP exposure induced dose-dependent adverse effects. Histopathological analysis revealed lung injury at 0.1 and 1 mg/kg/day, characterized by inflammatory cell infiltration and alveolar damage, which was corroborated by significantly elevated pathological scores (Figure 2C,D). Consistently, TCIPP exposure resulted in a marked increase in inflammatory cell counts, MLI, DI, airway wall thickness, and airway wall area, indicating enhanced inflammation, alveolar enlargement, parenchymal destruction, and airway remodeling (Figure 2E–I). Together, these findings demonstrate that TCIPP induces dose-dependent lung injury in male mice at both histological and quantitative levels.

**Figure 3:**
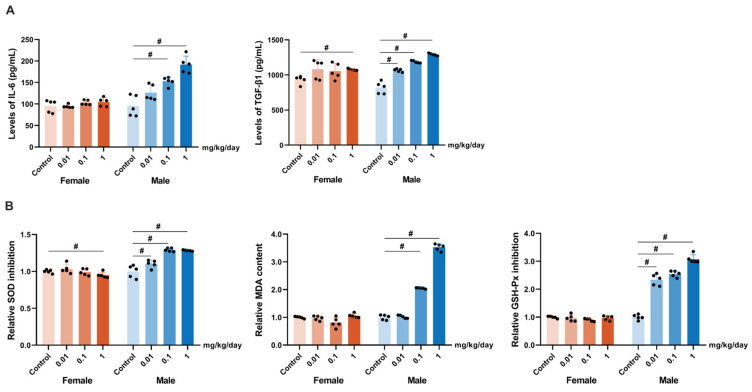
TCIPP exposure induces inflammatory responses and oxidative stress in mice (*N* = 5 per group). (**A**) Levels of IL-6 and TGF-β1 in lung tissues. (**B**) Normalized oxidative stress–related parameters, including SOD activity inhibition, MDA, and GSH-Px activity inhibition, in lung tissues. TCIPP, Tris(2-chloroisopropyl) phosphate; IL-6, pro-inflammatory cytokine; TGF-β1, transforming growth factor-beta 1; SOD, superoxide dismutase; MDA, malonydialdehyde; GSH-Px, glutathione peroxidase. Data are expressed as mean ± SEM; # *p* < 0.05, determined by one-way ANOVA followed by Tukey’s multiple comparisons test.

Furthermore, TCIPP exposure significantly increased levels of the inflammatory markers IL-6 and TGF-β1 in lung tissue (Figure 3A). Consistent with an oxidative stress mechanism, TCIPP also caused a significant inhibition of SOD and GSH-Px activities and an increase in MDA content in the lungs of male mice (Figure 3B). In contrast, female mice displayed no significant changes in lung pathology, IL-6 levels, GSH-Px activity, or MDA content across all doses. The only significant changes observed in females were elevated TGF-β1 and decreased SOD activity at the highest dose (1 mg/kg/day, Figure 3A,B).

In summary, TCIPP exposure induced dose-dependent lung injury, inflammation, and oxidative stress, with male mice showing greater sensitivity. Based on the significant alterations observed at 0.1 mg/kg/day in males, we further chose 0.1 mg/kg/day (TCIPP) and male mice for subsequent experiments.

### 3.4. Protective Effect of Niacin Against TCIPP-Induced Lung Injury

**Figure 4:**
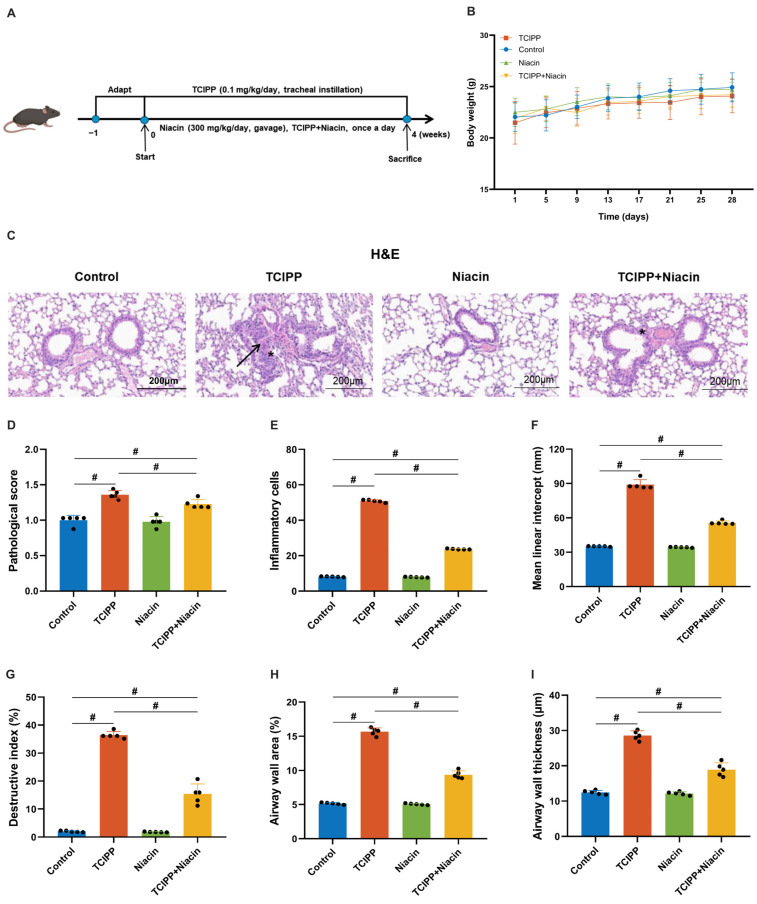
Niacin partially attenuates TCIPP-induced lung injury in male mice (*N* = 5 per group). (**A**) Experimental timeline of TCIPP exposure (0.1 mg/kg/day, intratracheal) and niacin supplementation (300 mg/kg/day, oral gavage) for 4 weeks. (**B**) Body weight changes in Control, TCIPP, Niacin, and TCIPP + Niacin groups. (**C**) Representative H&E-stained lung sections from each group, arrow (→) indicated alveolar wall thickening, inflammatory infiltration, and fibrin deposition, asterisk (*) marks hemorrhagic exudation. (**D**) Pathological scores of lung tissues. (**E**) Inflammatory cell counts. (**F**) Mean linear intercept. (**G**) Destructive index. (**H**) Airway wall area. (**I**) Airway wall thickness. TCIPP, Tris(2-chloroisopropyl) phosphate. Data are expressed as mean ± SEM; # *p* < 0.05, determined by one-way ANOVA followed by Tukey’s multiple comparisons test.

The niacin intake can promote the lung function level of participants. We further investigated whether niacin supplementation could decrease the levels of TCIPP-induced lung damage in male mice. Male mice models were exposed to TCIPP (0.1 mg/kg/day, intratracheal instillation) with or without niacin supplementation (300 mg/kg/day, oral gavage) for 4 weeks (Figure 4A). No significant differences in body weight were observed among groups (Figure 4B). Histological analysis revealed marked lung injury in the TCIPP group, including alveolar wall thickening, inflammatory infiltration, and structural disruption, which were significantly alleviated by niacin co-administration, also reducing pathological scores (Figure 4C,D). Quantitative analyses further supported these observations, showing that TCIPP exposure significantly increased inflammatory cell counts, MLI, DI, airway wall area, and airway wall thickness, all of which were partially but consistently reversed by niacin supplementation (Figure 4E–I).

**Figure 5:**
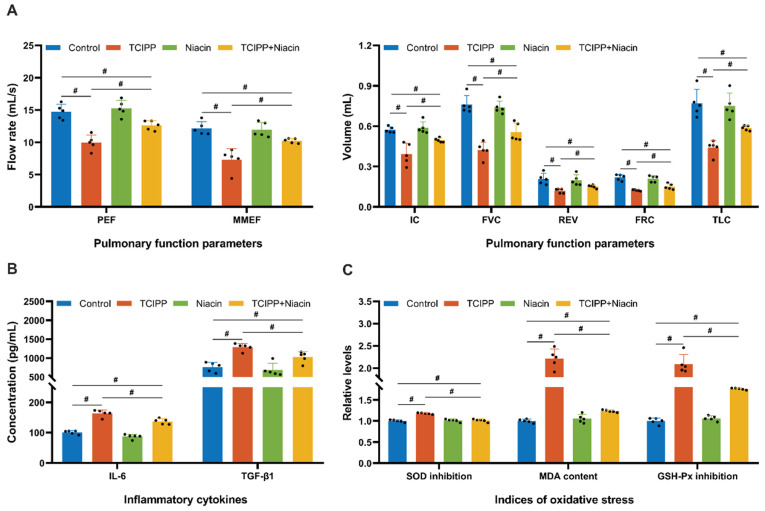
Niacin partially attenuates TCIPP-induced decline in lung function, inflammation, and oxidative stress in male mice (*N* = 5 per group). (**A**) Lung function parameters, including flow rate–related indices (PEF and MMEF; mL/s) and lung volume-related indices (IC, FVC, REV, FRC, and TLC; mL). (**B**) Levels of IL-6 and TGF-β1 in lung tissues. (**C**) Normalized oxidative stress–related parameters, including SOD activity inhibition, MDA, and GSH-Px activity inhibition, in lung tissues. TCIPP, Tris(2-chloroisopropyl) phosphate; PEF, peak expiratory flow; MMEF, maximal mid-expiratory flow; IC, inspiratory capacity; FVC, forced vital capacity; REV, residual volume; FRC, functional residual capacity; TLC, total lung capacity; IL-6, pro-inflammatory cytokine; TGF-β1, transforming growth factor-beta 1; SOD, superoxide dismutase; MDA, malonydialdehyde; GSH-Px, glutathione peroxidase. Data are expressed as mean ± SEM; # *p* < 0.05, determined by one-way ANOVA followed by Tukey’s multiple comparisons test.

Lung function tests demonstrated that TCIPP exposure significantly affected the PEF, MMEF, IC, FVC, REV, FRC, TLC, and Niacin supplementation partially modulated these lung function indicators compared to the TCIPP-exposed group (Figure 5A). Consistently, TCIPP exposure was elevated. Niacin supplementation significantly reduced the levels of the inflammatory cytokines IL-6 and TGF-β1 (Figure 5B). In parallel, niacin markedly alleviated TCIPP-induced oxidative stress, as evidenced by increased activities of SOD and GSH-Px, along with a decreased level of MDA in lung tissues (Figure 5C).

### 3.5. Niacin Attenuates TCIPP-Induced Lung Injury via the NF-κB Pathway

**Figure 6:**
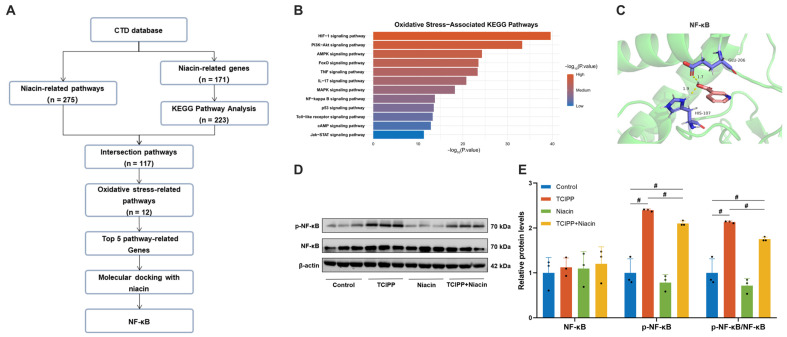
Identification and validation of niacin-targeted antioxidant pathways and key molecules. (**A**) Flowchart outlining the identification of key antioxidant pathways and molecules of niacin through the CTD, KEGG pathway analysis, and molecular docking analysis. (**B**) KEGG pathway analysis revealed the top oxidative stress-associated pathways linked to niacin. (**C**) Molecular docking interactions between niacin and the target proteins NF-κB. (**D**) Western blot analysis displaying protein levels of NF-κB and phospho-NF-κB (p-NF-κB) in lung tissues from Control, TCIPP, Niacin, and TCIPP + Niacin groups. (**E**) Quantification of relative protein levels. Data are presented as mean ± SEM; # *p* < 0.05, determined by one-way ANOVA followed by Tukey’s multiple comparisons test.

To investigate the effect mechanisms of niacin on TCIPP-induced lung damage, the CTD was utilized to identify niacin-related genes and pathways. Among 275 niacin-related pathways, 171 unique genes were identified. KEGG pathway analysis revealed 223 pathways associated with these genes, with 117 intersection pathways, and 12 directly related to oxidative stress (Figure 6A,B). Finally, the top 5 genes related to oxidative stress pathways were manually included (Table S4). Molecular docking analysis demonstrated that niacin has the highest binding affinity to the NF-κB, a binding energy of −4.48 kcal/mol, interacting primarily with HIS107, followed by AKT1 (−4.35 kcal/mol), MAPK3 (−4.33 kcal/mol), MAPK1 (−4.09 kcal/mol), and RELA (−3.96 kcal/mol, Figure 6C and Figure S3). To validate these findings, Western blot analysis revealed that niacin treatment significantly inhibited NF-κB phosphorylation in lung tissue (Figure 6D,E). Overall, niacin mitigated TCIPP-induced lung injury primarily by modulating the NF-κB pathway.

## 4. Discussion

Our findings indicated that dietary niacin was associated with mitigate TCIPP-induced lung function decline, with a more pronounced association observed in males. These findings were supported by the TCIPP-exposed mice model, where lung injury was more pronounced in male mice compared to females. Furthermore, niacin supplementation significantly attenuated lung damage in male mice, accompanied by reduced inflammation and oxidative stress, suggesting a potential modulatory role of niacin in pollutant-related lung injury, possibly involving the NF-κB signaling pathway.

The association between exposure to TCIPP and its metabolite BCIPP and adverse respiratory outcomes has been extensively investigated in previous studies [39]. At the cellular level, TCIPP exposure has been shown to induce cytotoxicity and apoptosis in lung epithelial cells, such as A549 cells, suggesting a direct toxic effect on lung tissue [20,21,40,41]. Epidemiologic studies further suggested that exposure to TCIPP was associated with impaired lung function [17,42,43] and an elevated risk of childhood respiratory conditions [44]. For instance, higher urinary BCIPP concentrations during pregnancy were associated with an increased risk of asthma and respiratory infections in offspring [42]. Additionally, cross-sectional and follow-up studies have also reported significant associations between TCIPP or BCIPP and a higher prevalence of asthma and allergic rhinitis [45,46,47,48]. These findings underscore the potential of TCIPP as a significant environmental risk factor for respiratory diseases, and the exploration of its pathogenic mechanisms and prevention strategies is critically important.

NHANES and animal studies reveal sex-specific differences in TCIPP-induced lung injury, matching prior evidence on pollutant vulnerability. Male mice are more susceptible to pollutant-induced mitochondrial and oxidative damage, while females are relatively resistant, possibly due to hormones [49,50]. Estrogen exhibits antioxidant and anti-inflammatory effects, including boosting antioxidant enzyme activity and inhibiting NF-κB signaling, thereby protecting tissues from toxic damage [51]. In this study, the reduced lung injury in female mice may have been further exacerbated by the uncontrolled estrous cycle. Estrogen level fluctuations are known to modulate redox balance and inflammation, with peak estrogen phases linked to increased antioxidant activity and decreased inflammatory signaling [52,53,54]. Future studies incorporating estrous cycle stratification or hormonal measurements will be crucial for precisely delineating sex-dependent mechanisms.

Dietary interventions, particularly the use of antioxidant vitamins such as vitamin C, E, D, and niacin, have important roles in preventing the harmful effects of environmental pollutants on pulmonary health [34]. Antioxidant vitamins exert protective effects by reducing oxidative stress and enhancing the antioxidant defenses, which are crucial in mitigating the adverse effects of pollutants like OPFRs [55]. The protective effects of niacin against lung damage are well-documented in the study, and niacin is recognized for its antioxidant and anti-inflammatory properties [56]. Niacin supplementation can reduce the pulmonary oxidative stress, such as malondialdehyde (MDA), and attenuate lung inflammation in mice [57,58]. Additionally, experimental evidence highlighted that niacin impaired antioxidant capacity, possibly through NF-κB signaling pathways [59]. As a precursor in the biosynthesis of nicotinamide adenine dinucleotide phosphate (NADPH), niacin helps supplement NAD^+^ and ATP levels depleted by oxidative stress, while also preventing GSH depletion [60,61]. In our animal models, niacin supplementation can significantly reduce TCIPP-induced lung toxicity by mediating the NF-κB pathway, which demonstrates niacin as an effective intervention against oxidative stress and inflammation caused by environmental pollutants. However, the niacin dose used in the animal model (300 mg/kg/day) represents a pharmacological exposure designed to elucidate mechanistic pathways within a short experimental period rather than to mimic human dietary intake. Based on standard body surface area-based dose conversion, 300 mg/kg/day in mice corresponds to a human equivalent dose of approximately 1.4–1.5 g/day, which falls within the pharmacological range previously reported to exert therapeutic effects in humans [62,63]. Nevertheless, future studies are needed to confirm physiologically relevant doses.

This study has several strengths, particularly employing the dietary intervention (niacin) as a strategy to alleviate TCIPP-induced lung damage, based on population studies and animal models. Furthermore, we identified the NF-κB pathway as a potential mechanism through which niacin exerts its protective effects on mice’s lung function. However, there are some limitations. Firstly, inclusion and exclusion criteria were used to ensure data quality, but individuals with severe lung function impairment in the NHANES cohort may not be able to obtain lung function data; older or less educated individuals may not be able to perform lung function procedures correctly, which can lead to selection bias in the inclusion of the population. It is possible that the relationship between pollutants and lung function has been underestimated. Second, although molecular docking, pathway enrichment, and protein expression analyses collectively suggest involvement of the NF-κB pathway, the direct functional validation of pathway specificity was not performed in this study, and the underlying molecular mechanisms remain to be fully elucidated in future studies.

## 5. Conclusions

In summary, this study provides integrated epidemiological and experimental evidence that niacin supplementation is associated with lung function in the TCIPP exposure, with sex-specific patterns observed. Niacin supplementation can alleviate inflammation and oxidative stress induced by TCIPP-related lung damage by modulating the NF-κB pathway. These findings advance a framework in which dietary nutrients may modify susceptibility to pollutant-related respiratory injury, highlighting the importance of considering nutrition and pollutant interactions in environmental health research.

## Figures and Tables

**Table 1:** Sex-specific distributions of urinary BCIPP, dietary niacin intake, and lung function indicators in NHANES 2011–2012.

Variables	All (*N* = 1031)	Male (*N* = 514)	Female (*N* = 517)	*p* Value
Urinary BCIPP concentration (µg/L), GM (GSD)	0.15 (2.55)	0.15 (2.45)	0.15 (2.65)	0.912
Urinary BCIPP concentration (ng/g creatinine), GM (GSD)	1.50 (2.65)	1.27 (2.48)	1.77 (2.74)	0.070
Dietary niacin intake (mg/d), mean ± SD	24.61 ± 12.87	28.75 ± 14.35	20.51 ± 9.59	<0.001
Lung function				
FEV_1_ (mL), mean ± SD	7.96 ± 0.31	8.13 ± 0.27	7.80 ± 0.26	<0.001
FVC (mL), mean ± SD	8.21 ± 0.29	8.39 ± 0.23	8.03 ± 0.23	<0.001
PEF (mL/s), mean ± SD	8.94 ± 0.31	9.11 ± 0.25	8.77 ± 0.26	<0.001
FEF_25–75%_ (mL/s), mean ± SD	7.85 ± 0.54	7.97 ± 0.57	7.73 ± 0.48	<0.001
FEV_1_/FVC, mean ± SD	0.58 ± 0.05	0.57 ± 0.05	0.58 ± 0.04	<0.001

NHANES, National Health and Nutrition Examination Survey; GM, geometric mean; GSD, geometric standard deviation; SD, standard deviation; FEV_1_, forced expiratory volume first second; FVC, forced vital capacity; PEF, peak expiratory flow; FEF_25–75%_, forced expiratory flow at 25–75% of FVC; BCIPP, bis(1-chloro-2-propyl) phosphate.

## Data Availability

The original contributions presented in this study are included in the article/Supplementary Material. Further inquiries can be directed to the corresponding author(s).

## Supplementary Materials

The following supporting information can be downloaded at: https://www.mdpi.com/article/10.3390/antiox15010085/s1, Figure S1: Flow diagram of the study population; Figure S2: Urinary BCIPP levels in mice following TCIPP exposure; Figure S3: Molecular docking interactions between niacin and the target proteins. Table S1: Concentrations and detection frequencies of urinary BCIPP (µg/L) in NHANES 2011—2012; Table S2: Baseline demographic characteristics of included subjects in NHANES 2011—2012; Table S3: Associations of BCIPP or dietary niacin intake with lung function; Table S4: Frequency of key genes across enriched KEGG pathways [64,65,66,67,68,69].
