# Supplementary material for: Niacin Supplementation Alleviates TCIPP-Induced Lung Injury via Inhibition of the NF-κB Signaling Pathway
Source: Antioxidants (Basel). 2026 Jan 8;15(1):85. doi: 10.3390/antiox15010085 (PMC12837827; doi:10.3390/antiox15010085)
Supplement: Supplementary file 1 [file antioxidants-15-00085-s001.zip › antioxidants-4036734-supplementary.pdf]

Supplementary Information

Supplementary Materials

Detection methods of urinary BCIPP in humans and mice

Covariates

The animal models

Hematoxylin and Eosin (H&E) Staining

Pulmonary Histopathological Scoring

Quantification of Inflammatory Cell Infiltration

Mean Linear Intercept (MLI)

Destruction Index (DI)

Airway Wall Thickness and Airway Wall Area

Enzyme-Linked Immunosorbent Assay (ELISA)

Determination of Oxidative Stress-related Indicators

Western blots

Supplementary Tables

Table S1. Concentrations and detection frequencies of urinary BCIPP (μg/L) in NHANES 2011—2012

Table S2. Baseline demographic characteristics of included subjects in NHANES 2011—2012

Table S3. Associations of BCIPP or dietary niacin intake with lung function

Table S4. Frequency of key genes across enriched KEGG pathways

Supplementary Figures

Figure S1. Flow diagram of the study population

Figure S2. Urinary BCIPP levels in mice following TCIPP exposure

Figure S3. Molecular docking interactions between niacin and the target proteins

Supplementary Materials

Detection methods of urinary BCIPP in humans and mice

Urine samples were collected from human individuals. 0.2 mL of urine was used for the enzymatic hydrolysis of BCIPP conjugates, followed by automated off-line solid-phase extraction, reversed-phase HPLC separation, and detection via isotope dilution-electrospray ionization tandem mass spectrometry. To ensure data reliability and accuracy, each analytical batch included high- and low-concentration quality control (QC) materials and reagent blanks. The concentrations of QC samples were evaluated using standard statistical quality control rules. Urine samples were collected from mice, mixed internal standards (10 ng of each) were added to 2 mL of urine, buffered with 0.7 mL of sodium acetate (pH = 5, 1M), and then digested with 100 μL of enzyme solution (1,000 units per mL, β-glucuronidase/arylsulfatase enzyme) at 37°C overnight. Samples were extracted using a StrataX-AW column with 2 mL of acetonitrile and 2 mL of water. After loading the sample, the filter was rinsed with 2 mL of water, and then the target chemical was eluted with 2 mL of 5% triethylamine acetonitrile. The eluate was concentrated to near dryness under a gentle stream of nitrogen, reconstituted with 1 mL of methanol, and filtered through a 0.22-micron nylon filter for instrumental analysis.

Covariates

For the sample-weighted multiple regression analyses between BCIPP or dietary niacin intake and lung function, a set of common potential confounders according to previous studies was adjusted[64]. Continuous covariates included age and log-transformed urinary creatinine, while categorical variables comprised sex (male/female), race (Mexican American, other Hispanic, non-Hispanic white, non-Hispanic black, non-Hispanic and other race), family poverty-income ratio (<1.00, 1.00-1.99, 2.00-3.99, ⩾4.00), guardian education level (less than 9th grade, 9-11th grade, high school graduate/GED or equivalent, some college or AA degree, college graduate or above), BMI categories (<25, 25-29.9, ⩾30 kg/m

^{2}), physical activity (never, moderate and vigorous), and serum cotinine levels (<0.05, 0.05-10, >10 ng/mL). All analyses accounted for the complex survey design through appropriate weighting.

The animal models

All mice were maintained under standardized conditions (12h light/dark cycle, 25±1 °C, 50-60% humidity) in the institutional animal facility and acclimatized for one week prior to experiments. The stock solution of TCIPP (20 mg/mL) was prepared by dissolving the standard in a mixture of 5% DMSO (Sigma, State of Missouri, USA), 30% PEG400 (MCE, Shanghai, China), 10% Tween 80 (Aladdin, Shanghai, China), and 55% physiological saline (Servicebio, Wuhan, China). The stock solution was stored at 4°C in the dark. For exposure, dilutions were made to achieve final concentrations of 0.2 mg/mL, 2 mg/mL, and 20 mg/mL, based on the appropriate dilution factors. Niacin was accurately weighed and dissolved in PBS (Servicebio, Wuhan, China), with constant stirring using a magnetic stirrer, until completely dissolved. The final concentration was 300 mg/mL.

Hematoxylin and Eosin (H&E) Staining

The left lung lobes were excised immediately after euthanasia. Residual blood was flushed with phosphate-buffered saline (PBS), followed by fixation in 4% paraformaldehyde for 24 hours at 4°C. Fixed tissues were subjected to graded dehydration, clearing, paraffin embedding, and sectioning. Prepared sections were stained with hematoxylin and eosin and examined under a pathological slide scanner.

Pulmonary Histopathological Scoring

Ten random microscopic fields per lung section were selected for evaluation. Lung tissue injury was semi-quantitatively scored based on inflammatory infiltration, edema, hemorrhage, and other pathological changes according to the following scale: 0: Normal lung architecture; 1: Pathological lesions occupying ≤ 25% of the total area; 2: Lesions occupying 26–50% of the

area; 3: Lesions occupying 51-75% of the area; 4: Lesions affecting > 75% of the area. Scores from all fields were averaged to obtain the final histopathological score for each sample.

Quantification of Inflammatory Cell Infiltration

Inflammatory cell infiltration in the lung parenchyma was quantified by counting the number of inflammatory cells in ten randomly selected fields per lung section. Results were expressed as the mean inflammatory cell count per field[65].

Mean Linear Intercept (MLI)

MLI was used to assess average alveolar diameter, as previously described[66, 67]. Briefly, within each randomly selected field, one horizontal and one vertical line were drawn through the central region, excluding blood vessels and airways. The total length of the lines and the number of alveolar septa intersecting them were recorded, and MLI was calculated as: MLI = total line length/number of alveolar septa.

Destruction Index (DI)

DI was used to evaluate the extent of alveolar structural destruction. DI was calculated as the percentage of destroyed alveoli relative to the total number of alveoli counted in each section[68].

Airway Wall Thickness and Airway Wall Area

Airway wall thickness and airway wall area were measured using Image-Pro Plus software[69]. The total airway area and luminal area were quantified, with wall area calculated as their difference. Airway wall thickness was determined by measuring the shortest and longest perpendicular distances between the luminal surface and the outer border of the airway smooth muscle layer.

Enzyme-Linked Immunosorbent Assay (ELISA)

Approximately 30 mg of lung tissue was weighed and homogenized in an appropriate volume of PBS using a tissue grinder to prepare lung tissue homogenates. The concentrations

of interleukin-6 (IL-6) and transforming growth factor-beta 1 (TGF-β1) in lung homogenates were quantitatively determined using commercial ELISA kits (Aifang Biology, Hunan, China) according to the manufacturer's instructions. These measurements were used to assess the degree of pulmonary inflammation.

Determination of Oxidative Stress-related Indicators

The levels of superoxide dismutase (SOD) activity, malonydialdehyde (MDA), and glutathione peroxidase (GSH-Px) activity in mice lung tissue were measured using commercial assay kits (Jiancheng Bioengineer, Nanjing, China) according to the provided protocols.

Western blots

By 10% sodium dodecyl sulfate-polyacrylamide gelelectrophoresis, equal amounts (60 μg) of protein were separated and transferred to nitrocellulose membranes (Millipore, Massachusetts, USA). Membranes were incubated with a 1:1000 dilution of antibodies for inducible NF-κB, P-NF-κB, or β-actin (Proteintech, Wuhan, China) for 2h at 37 °C. Finally, it was detected by ECL reagents (BIO-RAD, California, USA), and the densities of bands were quantified by Image J software.

Supplementary Tables

Table S1. Concentrations and detection frequencies of urinary BCIPP (μg/L) in NHANES 2011—2012

N	LOD	DF (%)	Min	25th	Median	75th	Max
1031	0.10	54.70	< LOD (0.07)	< LOD (0.07)	0.12	0.25	17.90

BCIPP, bis(1-chloro-2-propyl) phosphate; NHANES, National Health and Nutrition Examination Survey; LOD, limit of detection; DF, detection frequency; min, minimum; max, maximum.

Table S2. Baseline demographic characteristics of included subjects in NHANES 2011—2012.

Variables	All (N = 1031)	Male (N = 514)	Female (N = 517)	*P* Value
Age, mean ± SD	46.99 ± 16.12	46.60 ± 16.58	47.37 ± 15.65	0.441
Race, N (%)				0.522
Mexican American	100 (9.70)	54 (10.51)	46 (8.90)	
Other Hispanic	107 (10.38)	53 (10.31)	54 (10.44)	
Non-Hispanic White	407 (39.48)	213 (41.44)	194 (37.52)	
Non-Hispanic Black	250 (24.25)	116 (22.57)	134 (25.92)	
Non-Hispanic Asian	137 (13.29)	62 (12.06)	75 (14.51)	
Other races, including multi-racial	30 (2.91)	16 (3.11)	14 (2.71)	
Family poverty-income ratio, N (%)				0.882
< 1.00	233 (22.60)	121 (23.54)	112 (21.66)	
1.00-1.99	253 (24.54)	121 (23.54)	132 (25.53)	
2.00-3.99	221 (21.44)	107 (20.82)	114 (22.05)	
≥ 4.00	266 (25.80)	136 (26.46)	130 (25.15)	
Missing	58 (5.63)	29 (5.64)	29 (5.61)	
Education level, N (%)				**0.034**
9-11th grade (Includes 12th grade with no diploma)	83 (8.05)	50 (9.73)	33 (6.38)	
Less than 9th grade	136 (13.19)	69 (13.42)	67 (12.96)	
High school graduate/GED or equivalent	202 (19.59)	112 (21.79)	90 (17.41)	
Some college or AA degree	302 (29.29)	132 (25.68)	170 (32.88)	
College graduate or above	273 (26.48)	137 (26.65)	136 (26.31)	
Missing	35 (3.39)	14 (2.72)	21 (4.06)	
BMI (kg/m^2), N (%)				**0.004**
<25	296 (28.71)	146 (28.40)	150 (29.01)	
25-29.9	338 (32.78)	194 (37.74)	144 (27.85)	
≥30	392 (38.02)	172 (33.46)	220 (42.55)	
Missing	5 (0.48)	2 (0.39)	3 (0.58)	
Physical activity, N (%)				**< 0.001**
Never	619 (60.04)	265 (51.56)	354 (68.47)	
Moderate	214 (20.76)	98 (19.07)	116 (22.44)	
Vigorous	198 (19.20)	151 (29.38)	47 (9.09)	
Serum cotinine (ng/mL), N (%)				**< 0.001**
<0.05	533 (51.70)	232 (45.14)	301 (58.22)	
0.05-10	215 (20.85)	104 (20.23)	111 (21.47)	
>10	247 (23.96)	165 (32.10)	82 (15.86)	
Missing	36 (3.49)	13 (2.53)	23 (4.45)	
Urinary creatinine (mg/dL), mean ± SD	127.67 ± 86.63	143.85 ± 89.29	111.58 ± 80.84	**< 0.001**

NHANES, National Health and Nutrition Examination Survey.

Table S3. Associations of BCIPP or dietary niacin intake with lung function.

Variables	Models	BCIPP			Dietary niacin intake		
		β (95% CI)	*P* value	P_{adj}	β (95% CI)	*P* value	P_{adj}
FEV_1	Model 1	-0.08(-0.13, -0.03)	**< 0.001**	**0.002**	0.06(0.04, 0.08)	**< 0.001**	**< 0.001**
	Model 2	-0.05(-0.10, 0.00)	**0.030**	0.083	0.04(0.02, 0.06)	**0.003**	**0.008**
	Model 3	-0.05(-0.10, 0.00)	**0.041**	0.106	0.03(0.00, 0.06)	**0.009**	**0.019**
FVC	Model 1	-0.06(-0.10, -0.02)	**0.007**	**0.011**	0.05(0.03, 0.07)	**< 0.001**	**< 0.001**
	Model 2	-0.04(-0.08, 0.00)	0.077	0.129	0.05(0.03, 0.07)	**< 0.001**	**< 0.001**
	Model 3	-0.03(-0.07, 0.01)	0.117	0.194	0.04(0.02, 0.06)	**< 0.001**	**< 0.001**
PEF	Model 1	-0.08(-0.13, -0.03)	**0.002**	**0.005**	0.07(0.04, 0.10)	**< 0.001**	**< 0.001**
	Model 2	-0.06(-0.11, -0.01)	**0.033**	0.083	0.03(0.00, 0.06)	**0.033**	0.056
	Model 3	-0.06(-0.11, -0.01)	**0.042**	0.106	0.03(0.00, 0.06)	0.073	0.110
$FEF_{25\text{-}75\%}$	Model 1	-0.12(-0.22, -0.02)	**0.018**	**0.022**	0.07(0.02, 0.12)	**0.011**	**0.020**
	Model 2	-0.07(-0.18, 0.04)	0.181	0.226	0.00(-0.05, 0.05)	0.982	0.981
	Model 3	-0.07(-0.18, 0.04)	0.179	0.223	-0.01(-0.07, 0.05)	0.850	0.911
FEV_1/FVC	Model 1	-0.01(-0.02, 0.00)	0.069	0.069	0.00(-0.01, 0.01)	0.316	0.365
	Model 2	-0.01(-0.02, 0.00)	0.296	0.296	0.00(-0.01, 0.01)	0.146	0.183
	Model 3	-0.01(-0.02, 0.00)	0.259	0.259	0.00(-0.01, 0.01)	0.116	0.158

Model 1: adjusted for age, sex, and race. Model 2: additionally adjusted for family poverty-income ratio, educational level, BMI, physical activity, serum cotinine, and urinary creatinine based on Model 1. Model 3: additionally adjusted for dietary niacin intake concentration or BCIPP based on Model 2. BCIPP, bis(1-chloro-2-propyl) phosphate; FEV1, forced expiratory volume first second; FVC, forced vital capacity; PEF, peak expiratory flow; FEF25−75%, forced expiratory flow at 25−75% of FVC. P_{adj} is a *P* value corrected by False Discovery Rate.

Table S4. Frequency of key genes across enriched KEGG pathways

KEGG	Related genes	Counts
HIF-1 signaling pathway;<br>PI3K-Akt signaling pathway;<br>AMPK signaling pathway;<br>FoxO signaling pathway;<br>TNF signaling pathway;<br>IL-17 signaling pathway;<br>MAPK signaling pathway;<br>NF-kappa B signaling pathway;<br>p53 signaling pathway;<br>Toll-like receptor signaling pathway;<br>cAMP signaling pathway;<br>Jak-STAT signaling pathway	AKT1	7
	MAPK1	7
	MAPK3	7
	NFKB1	7
	RELA	7
	IL6	6
	IL1B	5
	TNF	5
	CASP3	4
	CASP8	4
	CCND1	4
	CDKN1A	4
	EGFR	4
	NFKBIA	4
	ATF4	3
	BCL2	3
	CDKN1B	3
	CXCL8	3
	EIF4EBP1	3
	KRAS	3
	MTOR	3
	PCK1	3
	PRKAA2	3
	PTGS2	3
	RPS6KB1	3
	TP53	3
	ANGPT1	2
	ANGPT2	2
	CASP9	2
	CCL2	2
	CCNB1	2
	CCNE1	2
	CEBPB	2
	FOXO1	2
	ICAM1	2
	IFNG	2
	NOS3	2
	SERPINE1	2
	SIRT1	2
	SLC2A4	2
	TEK	2
	TGFB1	2
	VCAM1	2
	VEGFA	2
	ADIPOQ	1
	BDNF	1
	CAT	1
	CD36	1
	CDK1	1

Continued Table S4. Frequency of key genes across enriched KEGG pathways

KEGG	Related genes	Counts
	DDIT3	1
	DUSP5	1
	ENO3	1
	F2R	1
	FASN	1
	GH1	1
	HIF1A	1
	HMOX1	1
	HSPB1	1
	KDR	1
	LEP	1
	LEPR	1
	LIPE	1
	NOS2	1
	NTRK2	1
	PARP1	1
	PPARG	1
	PPARGC1A	1
	SOD2	1
	TFRC	1
	VTN	1

Supplementary Figures

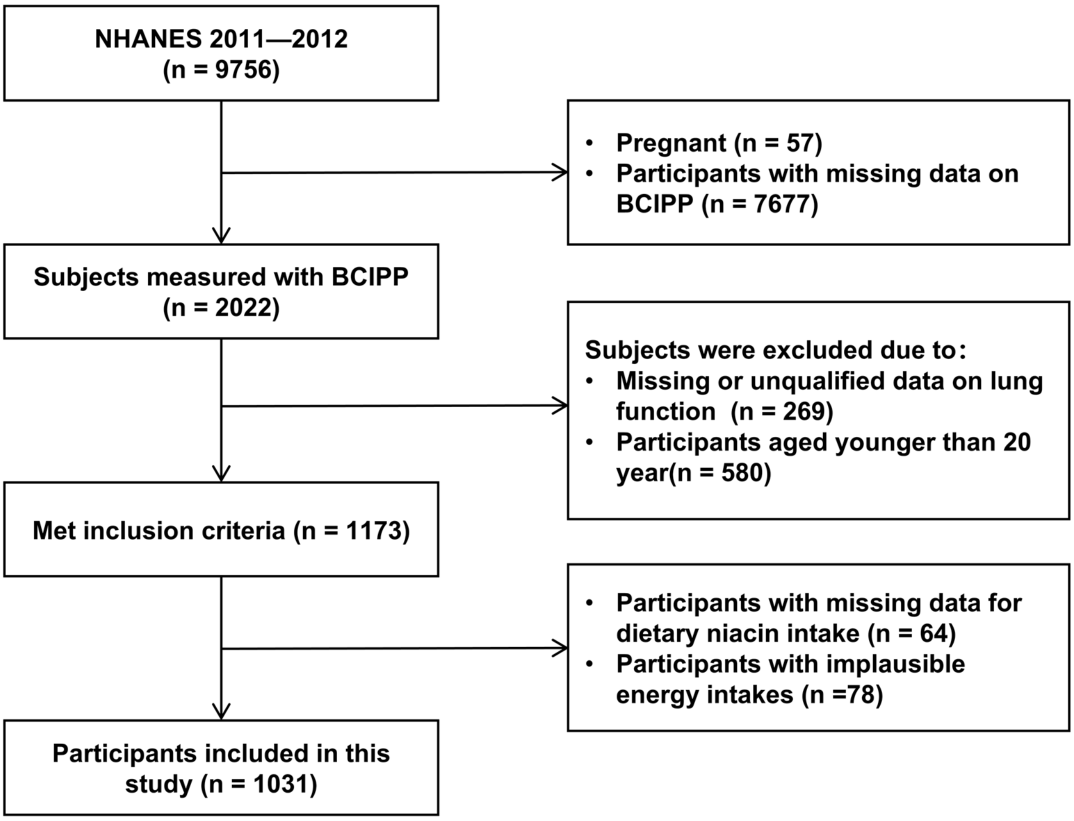

Figure S1. Flow diagram of the study population. NHANES, National Health and Nutrition Examination Survey; BCIPP, bis(1-chloro-2-propyl) phosphate.

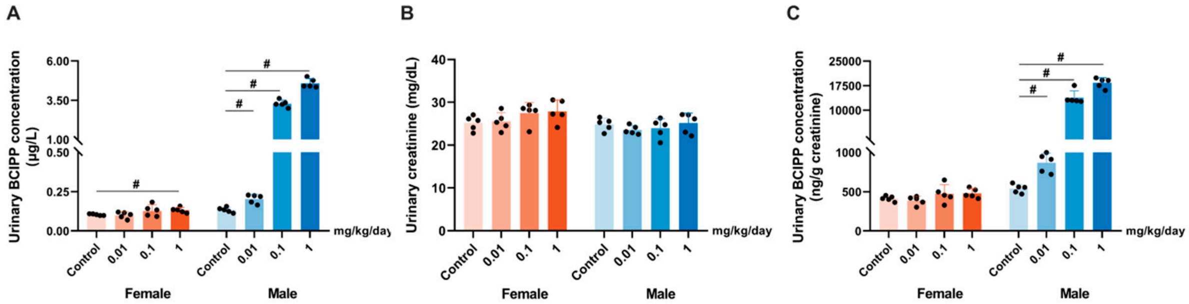

Figure S2. Urinary BCIPP levels in mice following TCIPP exposure (N = 5 per group). (A) Urinary BCIPP concentration (μg/L). (B) Urinary creatinine concentration (mg/dL). (C) Creatinine-adjusted urinary BCIPP concentration (ng/g creatinine). TCIPP, Tris(2-chloroisopropyl) phosphate. Data are expressed as mean ± SEM; #*P* < 0.05, determined by one-way ANOVA followed by Tukey's multiple comparisons test.

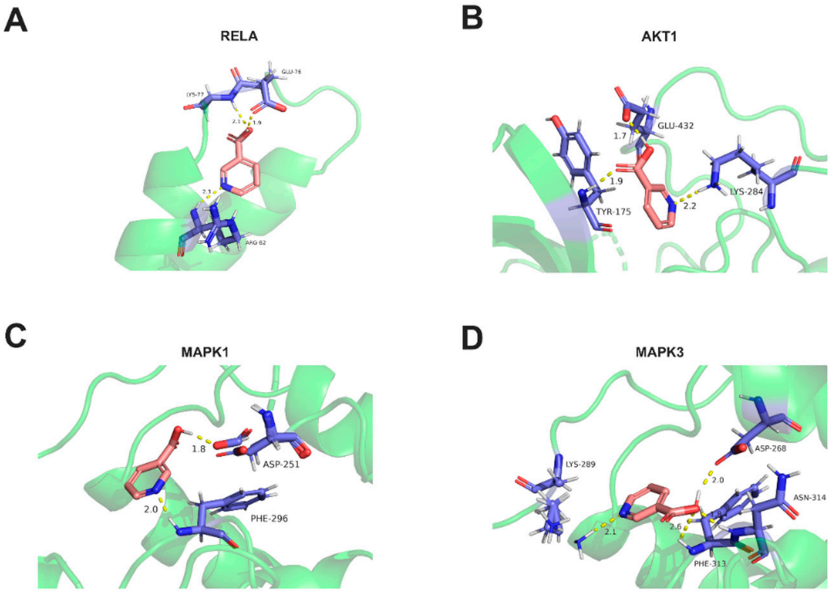

Figure S3. Molecular docking interactions between niacin and the target proteins. (A) RELA, (B) AKT1, (C) MAPK1, and (D) MAPK3.
